# Genome-wide transcriptome profiling reveals molecular response pathways of *Trichoderma harzianum* in response to salt stress

**DOI:** 10.3389/fmicb.2024.1342584

**Published:** 2024-02-01

**Authors:** Qihong Yang, Zhenchuan Mao, Yali Hao, Shijie Zheng, Jianlong Zhao, Yan Li, Yuhong Yang, Bingyan Xie, Jian Ling, Yanlin Li

**Affiliations:** ^1^College of Horticulture, Engineering Research Center for Horticultural Crop Germplasm Creation and New Variety Breeding (Ministry of Education), Hunan Mid-Subtropical Quality Plant Breeding and Utilization Engineering Technology Research Center, Hunan Agricultural University, Changsha, China; ^2^State Key Laboratory of Vegetable Biobreeding, Institute of Vegetables and Flowers, Chinese Academy of Agricultural Sciences, Beijing, China; ^3^School of Biological Sciences, Nanyang Technological University, Singapore, Singapore

**Keywords:** genome, metabolic, NaCl, salt tolerance mechanism, transcriptomic

## Abstract

*Trichoderma harzianum* exhibits a strong biological control effect on many important plant pathogens, such as *Fusarium oxysporum*, *Botrytis cinerea*, and *Meloidogyne*. However, its biocontrol effectiveness is weakened or reduced under salt stress. The aim of this study was to investigate the molecular response of *T. harzianum* to salt stress at the whole-genome level. Here, we present a 44.47 Mb near-complete genome assembly of the *T. harzianum* qt40003 strain for the first time, which was assembled *de novo* with 7.59 Gb Nanopore sequencing long reads (~170-fold) and 5.2 Gb Illumina short reads (~116-fold). The assembled qt40003 genome contains 12 contigs, with a contig N50 of 4.81 Mb, in which four of the 12 contigs were entirely reconstructed in a single chromosome from telomere to telomere. The qt40003 genome contains 4.27 Mb of repeat sequences and 12,238 protein-coding genes with a BUSCO completeness of 97.5%, indicating the high accuracy and completeness of our gene annotations. Genome-wide transcriptomic analysis was used to investigate gene expression changes related to salt stress in qt40003 at 0, 2% (T2), and 4% (T4) sodium chloride concentrations. A total of 2,937 and 3,527 differentially expressed genes (DEGs) were obtained under T2 and T4 conditions, respectively. GO enrichment analysis showed that the T2-treatment DEGs were highly enriched in detoxification (*p* < 0.001), while the T4 DEGs were mainly enriched in cell components, mostly in cellular detoxification, cell surface, and cell wall. KEGG metabolic pathway analysis showed that 91 and 173 DEGs were significantly enriched in the T2 and T4 treatments, respectively (*p* < 0.01), mainly in the glutathione metabolism pathway. We further experimentally analyzed the differentially expressed glutathione transferase genes in the glutathione metabolic pathway, most of which were downregulated (13/15). In addition, we screened 13 genes related to active oxygen clearance, including six upregulated and seven downregulated genes, alongside five fungal hydrophobic proteins, of which two genes were highly expressed. Our study provides high-quality genome information for the use of *T. harzianum* for biological control and offers significant insights into the molecular responses of *T. harzianum* under salt-stress conditions.

## 1 Introduction

Soil salinization, the excessive accumulation of salt and alkaline substances in the soil, is one of the major soil degradation threats occurring in the world, leading to an increase in soil pH and salt content, which limits the growth and development of plants. This is a common phenomenon across the globe, especially in arid regions and areas where irrigated agriculture is frequent. Human activities, such as over-reclamation of land, improper irrigation, and large-scale use of chemical fertilizers, have aggravated the problem of soil salinization for a long time, seriously affecting agricultural production, the ecological environment, and human welfare. At present, 0.8–3.6% of soils have become saline globally, about 1,125 million hectares of land are under threat of salinity, and 1.5 million hectares of land are becoming useless for agricultural production (Hossain and Shah, 2019). China’s saline–alkali land area is currently about 3.5 × 10^7^ hm^2^ and still increasing (Wang et al., 2011), accounting for 4.88% of the country’s arable soil area and mainly distributed in the northeast, north, northwest inland areas, and the coastal areas north of the Yangtze River (Zhang, 2020). Poor seed germination, withering of leaves, and, in severe cases, plant death are the signs of reduced water availability and osmotic pressure in saline soil (Kundu et al., 2022). Therefore, the adoption of effective measures to alleviate the harm of saline–alkali land to plants is an important challenge facing world agriculture.

*Trichoderma* spp. are widely found in nature, primarily in soil. They are one of the most widely used microbes for the biocontrol of plant diseases, and alter the response of plants to abiotic stresses (Zaidi et al., 2014). *T. harzianum* (*TH*), a *Trichoderma* species with great biocontrol potential, has good research value and broad research prospects. *TH* mainly acts on the resistance response of plants through competition, hyperparasitism, antibiosis, growth promotion, induction of plant resistance, and other aspects. Hashem et al. studied the effect of *TH* inoculation on *Ochradenus baccatus* in saline subsurface at soil concentrations of 0, 75, and 150 mM (Hashem et al., 2014), and showed that *TH* can increase the seed germination rate, dry weight of roots and aboveground parts, water content, membrane stability, chlorophyll content, total lipid content, and neutral lipid content of *Ochradenus baccatus* under salt stress, improving the antioxidant capacity and nutrient absorption capacity of plants. Umber et al. studied the effects of *TH* coating on wheat germination and seedling development under salt stress [60 and 120 mM sodium chloride (NaCl)] (Umber et al., 2021). The results showed that *TH* seed coating reduced the amount of hydrogen peroxide, catalase, and malondialdehyde and increased the protein content, ascorbate peroxidase, and total phenolics under salt stress, suggesting that its use is effective in the cultivation of crops in saline areas because it inhibits oxidative damage by triggering various phenolic compounds and scavenging proteins. Ahmad et al. studied the effects of different concentrations of NaCl (0, 100, and 200 mM) on factors including growth, physiobiochemical attributes, antioxidant enzymes, and oil content (Ahmad et al., 2015). Their results show that *TH* mitigated the detrimental effects of NaCl stress in mustard seedlings. Both enzymatic (SOD, POD, CAT, GR, APX, MDHAR, DHAR, GST, GPX), and non-enzymatic (ASA, GSH, GSSG) antioxidants are induced by NaCl, and *TH* further enhanced the synthesis of these phytoconstituents and protected the Brassica plants from further damage. However, the biocontrol effects of *TH* are restricted by various factors, such as chemical pesticides, soil salinization, environmental humidity, and temperature. Xiang et al. found that, as the sodium stress concentration of *TH* increased, the growth rate of the colony slowed down, the colony thinned, and the biomass decreased (Xiang et al., 2019). Nagarajan et al. found that an appropriate salt concentration can promote the metabolism of mycorrhizal fungi cells (Nagarajan and Natarajan, 1999). However, a high salt content affects the metabolism of cellular substances and even inhibits the growth of mycelia. The capacity of *Trichoderma* for biological disease control and environmental restoration depends on whether the strain has a high level of stress resistance (Vinale et al., 2008). In summary, while numerous studies have demonstrated that *TH* alleviates plant damage under salt stress, few have focused on elucidating the fungus’s own salt-tolerant mechanisms.

In this study, whole-genome sequencing and RNA sequencing (RNA-seq) technology were used to analyze the differential transcripts of *TH* strain qt40003 under normal conditions and NaCl stress, and to screen for the functional genes that regulate *TH* salt tolerance. The results of this study provide a theoretical basis for exploring the salt tolerance mechanism of *TH*, and have important relevance to making full use of the salt tolerance potential of *TH* and optimizing its application in sustainable agriculture in salinized areas.

## 2 Materials and methods

### 2.1 Materials

*T. harzianum* (*TH*) strain qt40003 was stored in the microbial preservation room of the Institute of Vegetables and Flowers, Chinese Academy of Agricultural Sciences (CAAS). The DNA extraction kit was procured from Sango Biotech Company, while the reverse transcription kit, RNA extraction kit, and qPCR kit were all obtained from TIAN GEN Biotech Company.

### 2.2 Methods

#### 2.2.1 Morphological and molecular identification of the strain qt40003

In order to determine whether the strain was *TH*, we removed the strain from the −80° refrigerator, picked it out with a vaccination needle and cultured it on PDA medium (28°C, out of light) for 5 days. Microhypha was picked up with tweezers and observed under a microscope. After spore production, spores were filtered with sterile water and four layers of lens paper to obtain a spore suspension, and then observed with a microscope.

The strains in question, namely qt40001, qt40003, and qt40443, were subjected to PCR amplification using the universal fungal primer *RPB2* (Saadaoui et al., 2023) (*RPB2*-5F2: 5′-GGGGWGAYCAGAAGAAGGC-3′, *RPB2*-7Cr: 5′-CCCATRGCTTGYTTRCCCAT-3′). The PCR amplification system was configured as follows: 1 μL of DNA template, 12.5 μL of 2× PCR Master Mix, 1 μL each of primers *RPB2*-5F2 and *RPB2*-7Cr, and 9.5 μL of ddH_2_O. The PCR amplification conditions were as follows: initial denaturation at 95°C for 3 min; followed by 35 cycles of denaturation at 95°C for 15 s, annealing at 56°C for 15 s, and extension at 72°C for 15 s; a final extension step at 72°C for 5 min. Subsequently, 5 μL of PCR amplification products were subjected to electrophoresis in 1 × TAE buffer solution, with DNA Marker serving as the control, and the PCR product bands were visualized using a gel imaging system.

Sequencing services were outsourced to Shenzhen BGI Co., LTD., and the obtained sequencing data were subjected to similarity analysis using Blast against the GenBank database. The clustering analysis was conducted using iqtree (v 2.0), the phylogenetic tree was constructed employing the Neighbor-Joining (NJ) method.

#### 2.2.2 Effects of NaCl stress on mycelial growth of qt40003

The strain was retrieved from the −80°C freezer and cultured at a constant temperature of 28°C without light for 3 days in Potato Dextrose Agar (PDA) medium (containing 200 g of potato, 20 g of glucose, 20 g of agar, and 1,000 mL of water). PDA media containing different concentrations of NaCl (0% as the control, 2, 4, 6, 8, and 10%) were prepared. Using a hole puncher (Φ = 6 mm), fungal cakes were removed from the edge of the activated colony and were then inoculated onto the center of the PDA plates with varying gradients. The plates were incubated at a constant temperature of 28°C without light. Colony morphology was recorded every 12 h. When the CK (0%) colonies occupied the entire culture dish, the colony diameters under different concentration gradients were measured, and the inhibitory rate of NaCl on colony growth was calculated as follows.


$$\begin{array}{c} \text{Colony growth}\\ \text{inhibition rate} \end{array}=\frac{\begin{array}{c} \text{CK colony diameter}-\text{colony diameter}\\ \text{after treatment} \end{array}}{\text{CK colony diameter}}\times 100\%$$

#### 2.2.3 Genome comparison

Using the nucmer module in MUMMER (v 3.23) with the parameter -maxmatch, a genome-wide comparison was made between IIPRTh-33 and qt40003 (Kurtz et al., 2004). The output of nucmer was filtered by delta-filtering (with the parameters -i 85-L1000-1 - r-q) to identify the one-to-one syntenic blocks between two genomes, visualize the results using the mummerplot module (with parameters -R IIPRTh-33.fa -Q qt40003.fa --filter --layout --large --png out.delt.filter > out1.png).

#### 2.2.4 Genome sequencing and assembly

The qt40003 genomic DNA extracted by the kit is sent to the Illumina (CA, United States) platform for sequencing. “k-mer analysis” is performed on the original data using Jellyfish (v2.2.3). When K value is 21, the peak of k-mer distribution curve is 24. The qt40003 genome size is estimated to be 40.79 Mb.

For third-generation sequencing, we used the native barcoding expansion kit (EXP-NBD104 and EXP-NBD114) and the ligation sequencing kit (SQK-LSK109) to prepare the library. Nanopore sequencing was performed on MinION (ONT R9.4.1). Reads generated on the Nanopore MinION platform were evaluated for sequence quality according to manufacturer’s protocol. Qt40003 generated a total of 7.59 Gb reads length for subsequent analysis.

We used canu (v 1.5) (Koren et al., 2017) and Nextdenovo (v 2.5.0) for genome *de novo* assembly. To improve assembly quality. We combined the two assembly results using QUICKMERGE (v 0.3) software. The genome was then polished using Racon (v 1.5.0) and Minimap2 (v 2.24), followed by 3 rounds of Pilon (parameters --changes --treads 6 --fix all) using second-generation Illumina’s short reads (Walker et al., 2014). A circular map of the genome was obtained using CIRCOS (v 0.69) (Krzywinski et al., 2009).

We conducted a genome evaluation using BUSCO (version 5.3.2, https://busco.ezlab.org/) with the following parameters: -i harzi_40,003.fa -lineage_dataset Fungi_odb10 -out output -m genome.

#### 2.2.5 Repeat content identification

We used homology and *de novo* methods to identify the repeated sequences in qt40003 genome. For the process of *de novo* methods, we used LTR-FINDER, REPEATSCOUT (Price et al., 2005), MITE-HUNTER (Han and Wessler, 2010) and PILER-DF (Edgar and Myers, 2005) to build the *de novo* repeat sequence library. For the process of the homology, we used Repbase database (v 20.05) (Bao et al., 2015). The final repeat sequence library is created from the *de novo* constructed database together with the Repbase database. Finally, we used REPEATMASKER (v 4.0.9) (Tarailo-Graovac and Chen, 2009) (http://repeatmasker.org/) to identify and classify the repeats in qt40003 genome. To detect species-specific repetitive elements, we employed repeatmodeler (v 2.0 with the following parameters: BuildDatabase -name contig.db -engine ncbi qt40003.fa.masked RepeatModeler -pa 10 -database contig.db > & RepeatModeler.log).

#### 2.2.6 Gene structure annotation

We employed three distinct approaches for the prediction of protein-coding genes: *de novo* prediction, gene prediction grounded in protein homology, and facilitated annotation leveraging RNA-seq data. The *ab initio* predictions were executed through the utilization of GENESCAN (Burge and Karlin, 1997), AUGUSTUS (v 2.7) (Stanke and Waack, 2003), GLIMMERHMM (v 3.0.4) (Majoros et al., 2004), GENEID (v 1.4) (Blanco et al., 2007), and SNAP (Korf, 2004). Gene prediction, based on homologous genes, was conducted employing GEMOMA (v 1.3) (Keilwagen et al., 2016) and entailed model training grounded in the coding sequence of *TH* CBS 226.95 (assembly Triha v1.0), as made available by NCBI. The RNA-seq reads of qt40003 were subjected to assembly via HISAT (v 2.0.4) (Kim et al., 2015) and STRINGTIE (v 1.2.3) (Pertea et al., 2015), subsequently facilitated by PASA (v 2.0.2) (Campbell et al., 2006) for elucidating unique i-genes, which contributed to the gene prediction process. Finally, we amalgamated the outcomes employing EVIDENCEMODELER (Haas et al., 2008).

#### 2.2.7 Gene functional annotation analysis

We uploaded the protein sequence of qt40003 to eggnog (v 2.1.12; http://eggnog-mapper.embl.de/) for comparison with some protein sequence databases to annotate gene function. Including Nr (NCBI verbose protein sequence) (Pruitt et al., 2007), Nt (NCBI non-redundant nucleotide sequence) (Bateman et al., 2004), KOG (Galperin et al., 2021) and Swiss-Prot (https://www.ebi.ac.uk/uniprot/).

#### 2.2.8 RNA-seq with different treatments

We employed a 6 mm diameter hole puncher to extract fungal cakes from the periphery of naturally growing 3d qt40003 mycelia. Each treatment was performed with three replicates to ensure the reliability of the results. Subsequently, these fungal cakes were individually inoculated onto Potato Dextrose Broth (PDB) medium containing 0, 2, and 4% concentrations of NaCl. The cultures were maintained at 28°C in the dark with shaking at 200 rpm/min. After a cultivation period of 3d, the mycelial samples were collected. Subsequently, these samples were submitted to Shanghai Personal Biotechnology Co., Ltd. for subsequent processing, including total RNA extraction, purification, quality assessment, and cDNA library construction. Using the sequencing by synthesis technology, which involves simultaneous synthesis and sequencing, the cDNA libraries were subjected to sequencing using the Illumina Hiseq XTen high-throughput sequencing platform.

#### 2.2.9 GO and KEGG annotation of differentially expressed genes

We employed topGO (Chen et al., 2019) to perform Gene Ontology (GO) enrichment analysis. Within this analysis, differentially expressed genes annotated with GO terms were utilized to compute the gene list and gene count for each specific term. Subsequently, we computed the *p*-value using the hypergeometric distribution method (with significance criteria set at *p*-value < 0.05) to assess whether the differential genes were significantly enriched within the GO terms, compared to the entire genomic background. This process facilitated the determination of the primary biological functions associated with the differentially expressed genes.

For the Kyoto Encyclopedia of Genes and Genomes (KEGG) enrichment analysis, we utilized clusterprofiler (v 4.0) (Wu et al., 2021). In this analysis, the differentially expressed genes annotated with KEGG pathways were employed to calculate the gene list and gene count for each individual pathway. We subsequently employed the hypergeometric distribution method to compute the *p*-value (with a significance threshold of *p*-value < 0.05) in order to identify KEGG pathways that exhibited significant enrichment of differentially expressed genes when contrasted with the overall genomic background.

#### 2.2.10 Analysis of differentially expressed genes under salt stress

The identification of significantly enriched metabolic pathways was achieved through an enrichment analysis of KEGG metabolic pathways. Subsequently, the expression levels of differentially expressed genes involved in these pathways were visualized as correlated heat maps using the R language. Multiple sequence comparisons of differentially expressed genes were performed using Muscle (v 3.8.31). The phylogenetic tree was constructed using IQTREE (v 2.2.5 with the following parameters: −nt 20, −m test, −b 1000).

#### 2.2.11 Validation of the RNA-seq results by qPCR

To assess the accuracy of RNA-seq data, we meticulously selected 10 genes exhibiting significant differential expression and designed primers using Primer (v 5.0) and SnapGene (v 2.3.2). Subsequently, we reverse-transcribed RNA from both the control (CK) and T4-treated (T4) samples into complementary DNA (cDNA) utilizing the PrimeScriptTM 1st strand cDNA Synthesis Kit. Utilizing the cDNA as a template and Tubulin (Montero-Barrientos et al., 2011) as the internal reference gene, we performed fluorescence quantification using the TB GreenTM Premix EX TaqTM fluorescence quantitative kit and QuantStudioTM real-time PCR software. Relative expression levels were determined utilizing the −ΔΔCt method, with each sample analyzed in triplicate for both biological and technical replicates.

## 3 Results

### 3.1 Morphological and molecular identification of the strain qt40003

**Figure 1:**
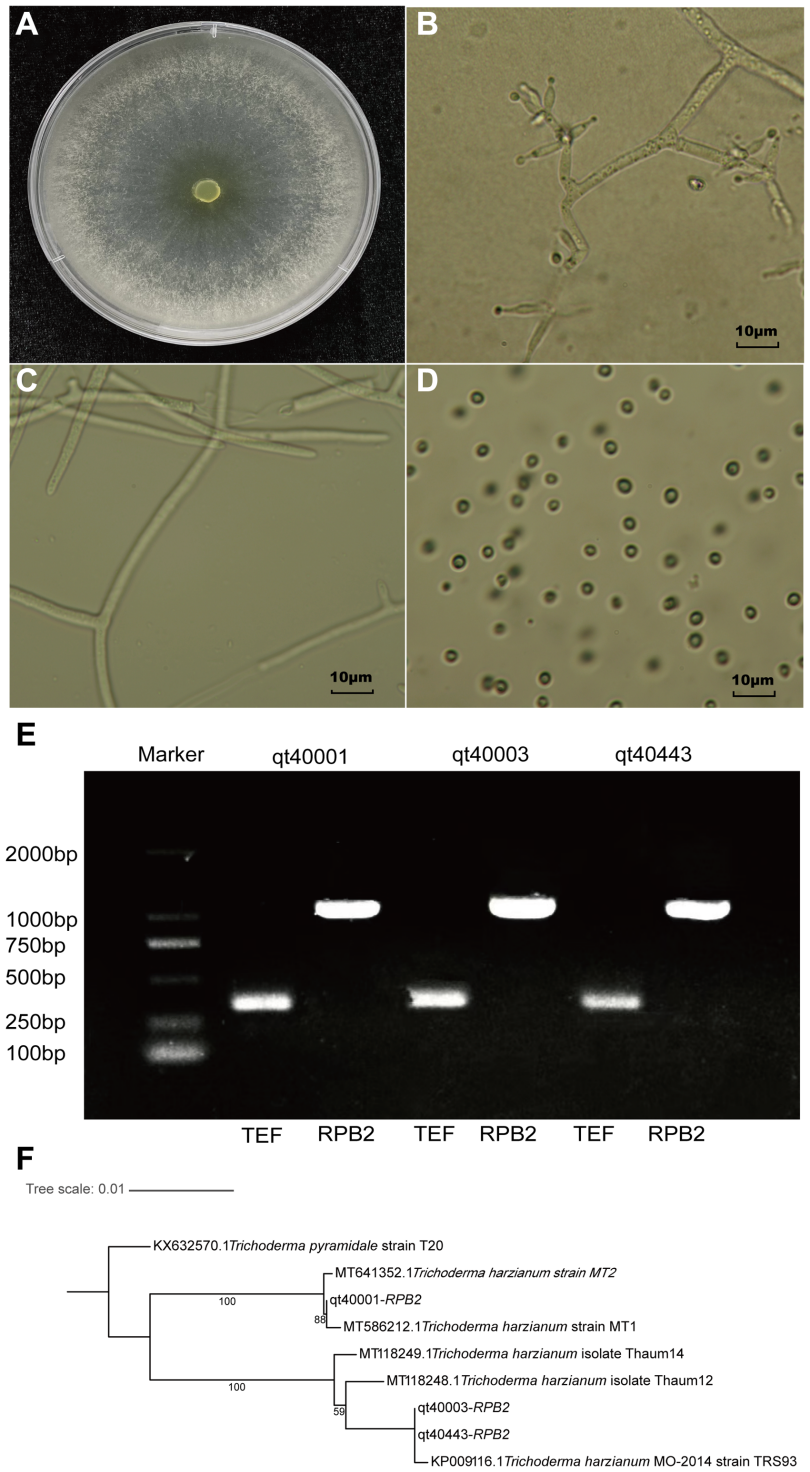
The morphological and molecular identification of qt40003. **(A)** The colony morphology of qt40003. **(B)** Microscopic observation of conidiophore. **(C,D)** Microscopic observation of mycelia and conidia. **(E)** PCR molecular identification was performed with specific primers. (Marker: Trans 2Kb DNA Marker, qt40001 and qt40443: other *Trichoderma harzianum* strains, *TEF*: Translation Elongation Factor and *RPB2*: RNA Polymerase II Second Largest Subunit). **(F)** Phylogenetic tree based on *RPB2* sequences of qt40003 and 8 other different *Trichoderma* species.

The *T. harzianum (TH)* strain qt40003 was derived from the Institute of Vegetable and Flower Research, Chinese Academy of Agricultural Sciences. The morphology of the strain was identified, and the characteristics of colony morphology, mycelia, and conidia were examined. The colony was white in the early stage of growth and turned yellow–green after spore formation. The strain consisted of colorless or light airborne mycelia and basal intracellular mycelia. The airborne mycelia were white, flocculent, dense, and concentric whorled (Figures 1A–C). The conidium was transparent ovoid (Figure 1D).

We further used the universal fungal primers *TEF* and *RPB2* for PCR-based molecular detection. We amplified DNA fragments of about 420 bp and 1,000 bp, respectively (Figure 1E). We input the amplified *RPB2* fragments into the BLAST search of the NCBI database. The *RPB2* sequence of this strain showed the highest consistency (99.89%) with the published *TH* MO-2014 strain. To analyze the evolutionary relationship of qt40003 in the *Trichoderma* genus, the *RPB2* sequences of qt40003 and eight other *Trichoderma* species were used to construct phylogenetic trees, and *T. pyramidate* was used as an outgroup (Figure 1F). Strain qt40003 was clustered with the MO-2014 strain, indicating the closest genetic distance between them. Furthermore, we constructed a phylogenetic tree using the *TEF* sequence of this strain and the top five *Trichoderma* species with the highest alignment rates in NCBI, with *T. pyramidate* as the outgroup. The closest genetic distance was between qt40003 and the *TH* TUB F-684 strain (Supplementary Figure S1). Based on morphological and molecular identification, this strain was confirmed as a *TH* strain.

### 3.2 Genome sequencing and assembly

We employed the Jellyfish tool to evaluate the genome size of qt40003 using Illumina sequence data (5.2 Gb, ~116-fold), based on the k-mer distribution (k-mer = 21). As a result, the estimated qt40003 genome size was approximately 40.79 Mb (Supplementary Figure S2).

The entire genome of strain qt40003 was comprehensively sequenced. For the Nanopore platform, we generated approximately 7.59 Gb of long reads for qt40003, equivalent to approximately 170-fold coverage. The genome assembly process requires separate assembly using NextDenovo and Canu (Koren et al., 2017), and the two assembly results were merged using QUICKMERGE. Subsequently, we enhanced the initial assembly using Racon (Xingxing et al., 2021), incorporating the Nanopore reads for refinement. Following correction with the Illumina short reads (5.2Gb), the genome size of the qt40003 genome final assembly was 44.5 Mb, consisting of 12 contigs The longest contig was 9.63 Mb, and the contig N50 value was 4.81 Mb (Table 1). To assess the assembly completeness, we remapped the Illumina sequences to the assembled qt40003 genome and 97.62% of reads could be mapped to the genome, indicating that the qt40003 assembly covered almost all the genomic region. Four of the 12 contigs (contigs 1, 2, 6, and 7) contained telomeric repeats—(5′-TTAGGG-3′)^n^ or (5′-CCCTAA-3′)^n^—at both contig ends, indicating that these contigs reached the telomere to telomere (T2T)-chromosome level.

**Table 1:** Statistics of the *Trichoderma harzianum* qt40003 strain genome assembly.

Feature	Statistics
Length of genome assembly (Mb)	44.47
Number of contigs	12
Max contig length (bp)	9,627,940
Contig N50 (bp)	4,809,098
Total length of genes (Mb)	21.89
Repetitive sequence	11,517
Gene number	12,238
GC	47%
Complete BUSCOs (C)	739 (97.5%)

**Figure 2:**
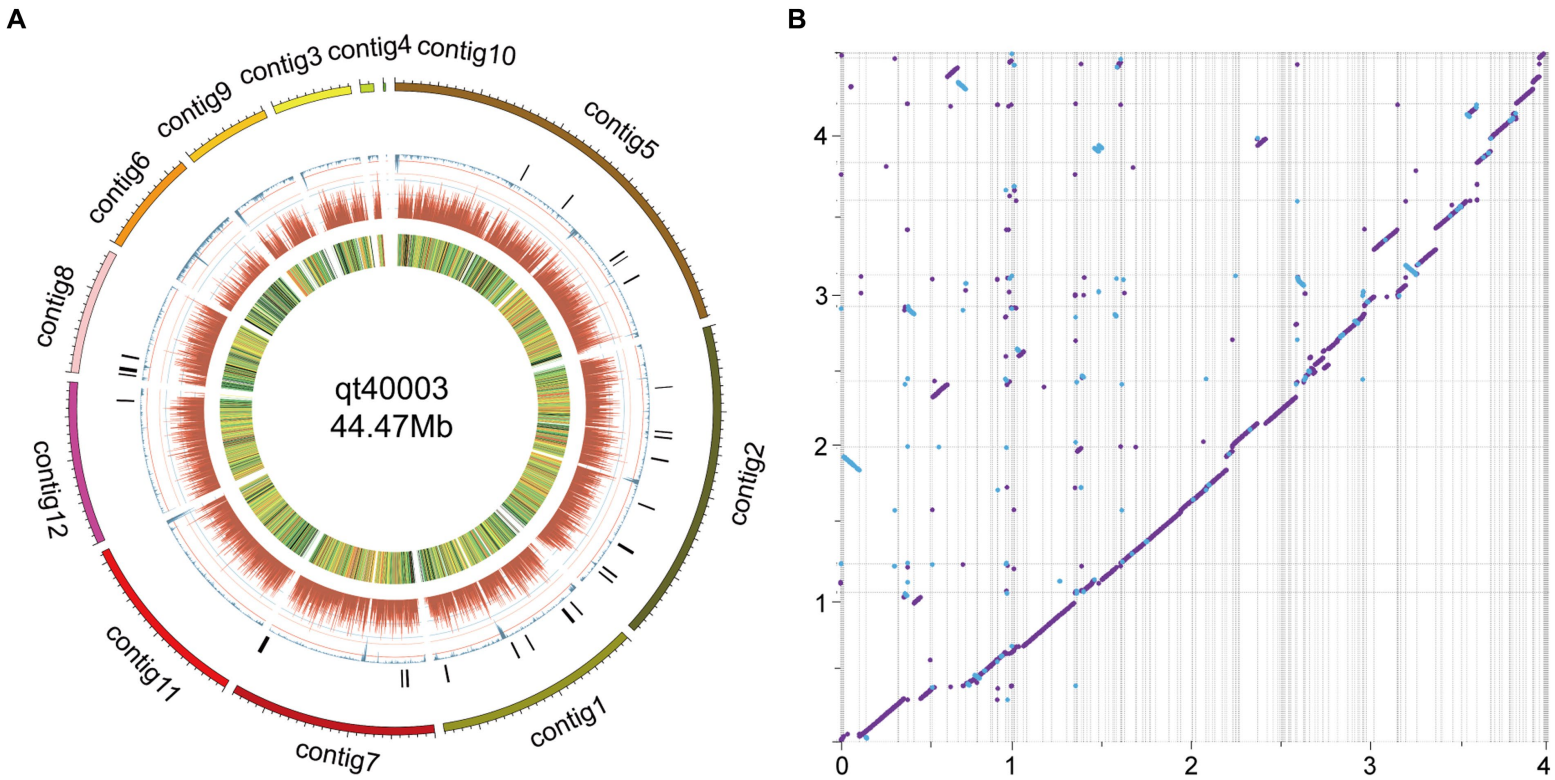
Genome features of qt40003 and comparative genomic analysis with published *T. harzianum* genome. **(A)** The outermost circle is the contigs. **(B)** The syntenic relationships between qt40003 and IIPRTh-33. Y-axis represents qt40003 contigs, and x-axis represents IIPRTh-33 genome. The axis tick values represent genome size (×10 Mb). The purple dot or line represents forward matches, and the blue dot or line represents reverse matches between two genomes.

On comparison with the published genome of the *TH* strain IIPRTh-33 (accession *SAMN22210987*; 456 contigs with N50 of 0.2 Mb), the two genomes displayed a highly collinear relationship, while some structural variations including inversions and translocations could also be detected (Figure 2B). Furthermore, we performed a comparative analysis with the genome of the *TH* strain CDMCC_20739 (accession *SAMN17621345*) at the chromosomal assembly level. The results (Supplementary Figure S3) demonstrated high collinearity between the chromosomes; however, significant structural variations, such as large-scale inversions and translocations, were evident.

We mapped the genome circle of qt40003 (Figure 2A). The outer-to-inner bar charts represent secondary metabolite gene clusters (in black), the repeat sequence density (in blue), gene density (in dark red), and gene expression levels from transcriptome data [in fragments per kilobase of transcript per million mapped reads (FPKM)].

### 3.3 Genome annotation and assembly assessment

Of the assembled qt40003 sequences, 9.61% (4.27 Mb) were annotated as repeating sequences (Supplementary Table S1). Of these, 4.89% were long terminal repeats (LTRs), and the three longest repeats were LTR/Gypsy (2.16 Mb), LINE/Tad1 (0.54 Mb), and DNA/MITE (96,367 bp). A total of 12,238 protein-coding genes were predicted for qt40003, of which 11,705 (95%) were supported by RNA-seq, and 9,986 (85%) were functionally annotated.

We used the KOG database to classify genes by orthologous lineation (Supplementary Figure S4). A total of 10,270 (87%) genes in the genome of strain qt40003 were annotated to corresponding functions. The most numerous functional classes were carbohydrate transport and metabolism (706), secondary metabolites biosynthesis, transport, and catabolism (679), and posttranslational modification, protein turnover, and chaperones (637), indicating that the strain had abundant carbohydrate metabolism. To assess the accuracy of the assembly, we performed a BUSCO (Benchmarking Universal Single-Copy Orthologs) evaluation of the qt40003 genome, and 739 (97.5%) gene models were complete, indicating that these combinations contained the majority of the qt40003 gene space (Supplementary Table S2).

### 3.4 Effects of different sodium NaCl on the growth of qt40003

**Figure 3:**
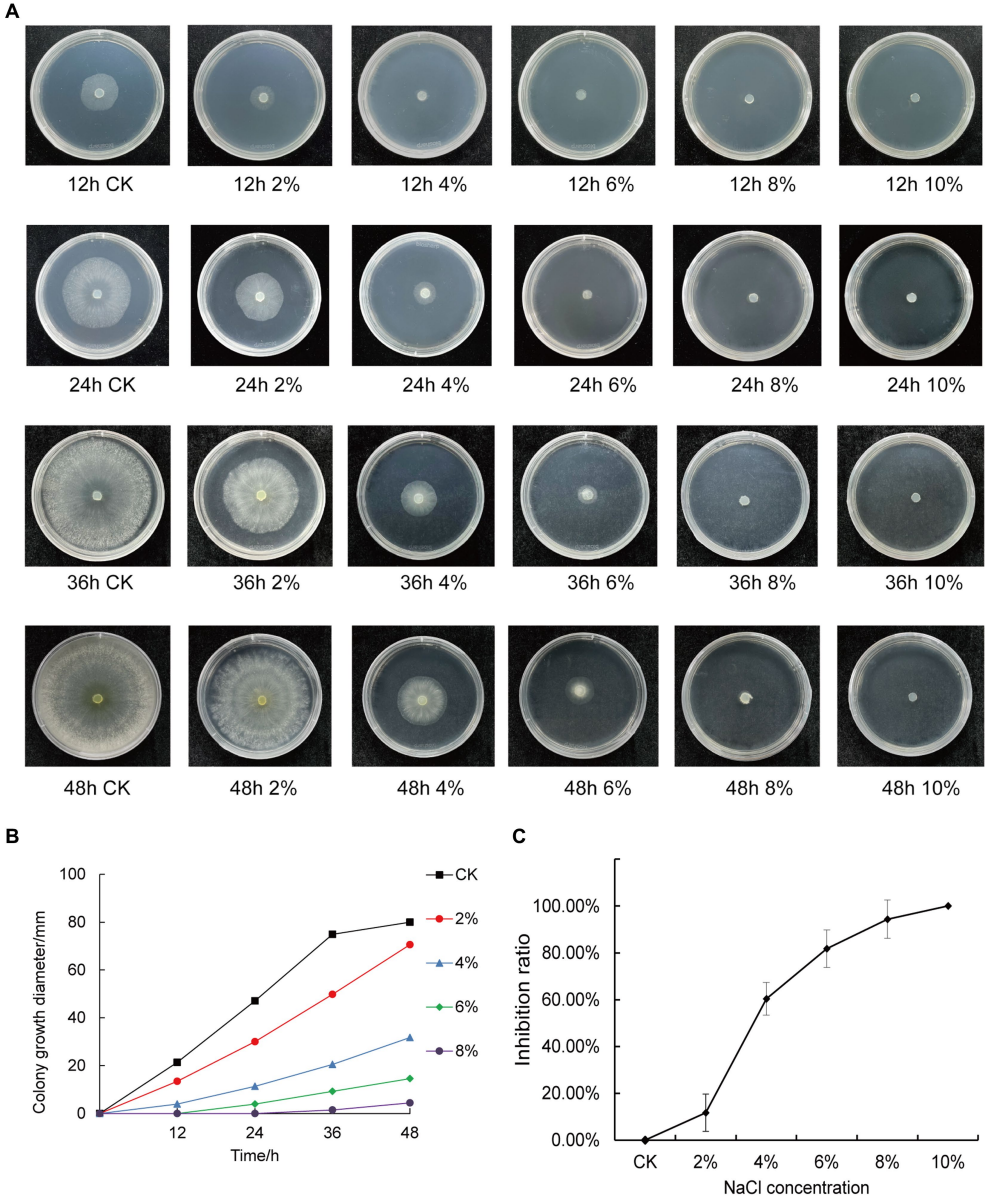
Effects of different concentrations of NaCl on the growth of qt40003. **(A)** 48 h growth of strain qt40003 on NaCl PDA. **(B)** Colony size at different NaCl concentrations. **(C)** Inhibition rate of different NaCl concentrations on the growth of strains. Error lines are standard errors.

We inoculated qt40003 on PDA medium with different NaCl concentrations and incubated it at 28°C for 48 h without light. Colony size was negatively correlated with NaCl concentration. The control group (CK) grew rapidly, the whole Petri dish was covered with mycelium in 48 h, and the mycelium was dense and wadded. However, as the NaCl concentration increased, the germination time of the qt40003 mycelium was delayed, the growth rate of the colony was significantly slowed, and the colony became thinner (Figure 3A). Under 10% NaCl stress (T10), mycelia did not germinate. The budding time of the CK, 2% (T2), and 4% (T4) NaCl groups was 12 h for mycelia to appear, while at 6% (T6) and 8% (T8) NaCl, mycelia appeared at 24 h and 36 h, respectively (Table 2). At 48 h, the inhibition rates of different NaCl concentrations on qt40003 colonies of CK, T2, T4, T6, T8, and T10 were 0, 11.75, 60.38, 81.75, 94.38, and 100%, respectively, and the overall growth curve for each treatment showed a slightly curved linear shape (Figure 3B). The inhibition rate curve exhibited a pronounced upward trend starting from T2, reaching 100% inhibition at T10 (Figure 3C).

**Table 2:** Effects of different NaCl stress on physiological indexes of 40,003 mycelia.

Concentration	Average daily height growth/mm	Hyphal diameter/mm	Mycelium budding time/h	Inhibition ratio
CK	25.00 ± 2.29	80 ± 0.00	12 h	0.00%
2%	17.66 ± 2.02	70.4 ± 2.61	12 h	11.75%
4%	7.83 ± 2.25	31.2 ± 2.03	12 h	60.38%
6%	3.50 ± 0.50	14.71 ± 2.00	24 h	81.75%
8%	1.1 ± 0.10	4.46 ± 2.10	36 h	94.38%
10%	/	/	/	100.00%

### 3.5 Transcriptome analysis of qt40003 under NaCl stress

The Illumina HiSeq X Ten platform was used for RNA-seq analysis of nine transcription samples from the CK, T2, and T4 groups. A total of 10.83 Gb of data were obtained, with an average of 3.61 Gb per sample, and the average map ratios were 99.29% for CK, 99.33% for T2, and 99.35% for T4 (Supplementary Table S3). Before performing differential expression analysis, the correlation of the gene expression levels between the samples was checked. We used the Pearson correlation coefficient, *r*, as the evaluation index for inter-sample correlation. The calculated *r* value was used to build a correlation heat map (Supplementary Figure S5). The results showed a high correlation between the samples, which can be used for further analysis.

DESeq was used for differential analysis of gene expression. A total of 2,937 differentially expressed genes (DEGs) were obtained from CK vs. T2, including 1,205 upregulated genes and 1732 downregulated genes. A total of 3,527 DEGs were obtained from CK vs. T4, including 1,484 upregulated genes and 2043 downregulated genes. The number of up- and downregulated genes in qt40003 both increased by 590 genes under a high NaCl concentration (T4) compared with a low NaCl concentration (T2). The comparison of DEGs between the two treatments revealed that the log_2_(fold change) of certain upregulated genes also increased with the rise in NaCl concentration.

**Figure 4:**
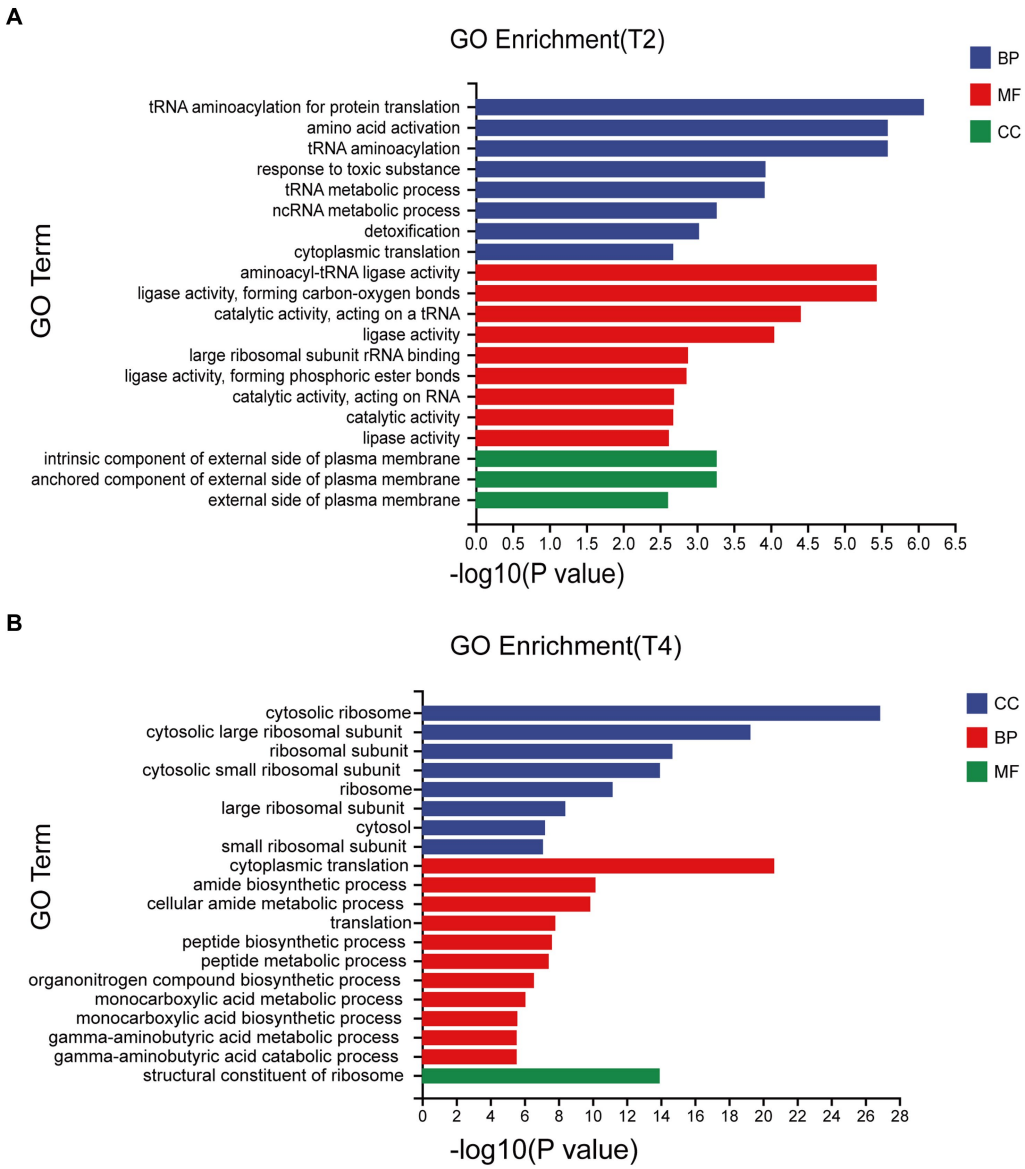
Go enrichment classification of differentially expressed genes under different treatments. **(A)** Go enrichment classification of differentially expressed genes treated with T2. **(B)** Go enrichment classification of differentially expressed genes treated with T4.

Significant enrichment analysis was performed on the RNA-seq data structures of the comparison groups treated with NaCl in the GO database (Gene Ontology, http://geneontology.org/), including three first-level classifications and 40 s-level classifications of biological processes (BP), molecular functions (MF), and cell components (CC). Of these, T2 accounted for the highest proportion of BP and T4 accounted for the highest proportion of CC. The DEGs involved in biological processes were mainly concentrated in tRNA aminoacylation for protein translation, amino acid activation, and tRNA aminoacylation, while the DEGs associated with cell components were mainly cytosolic ribosome and the cytosolic large ribosomal subunit (Figure 4).

**Figure 5:**
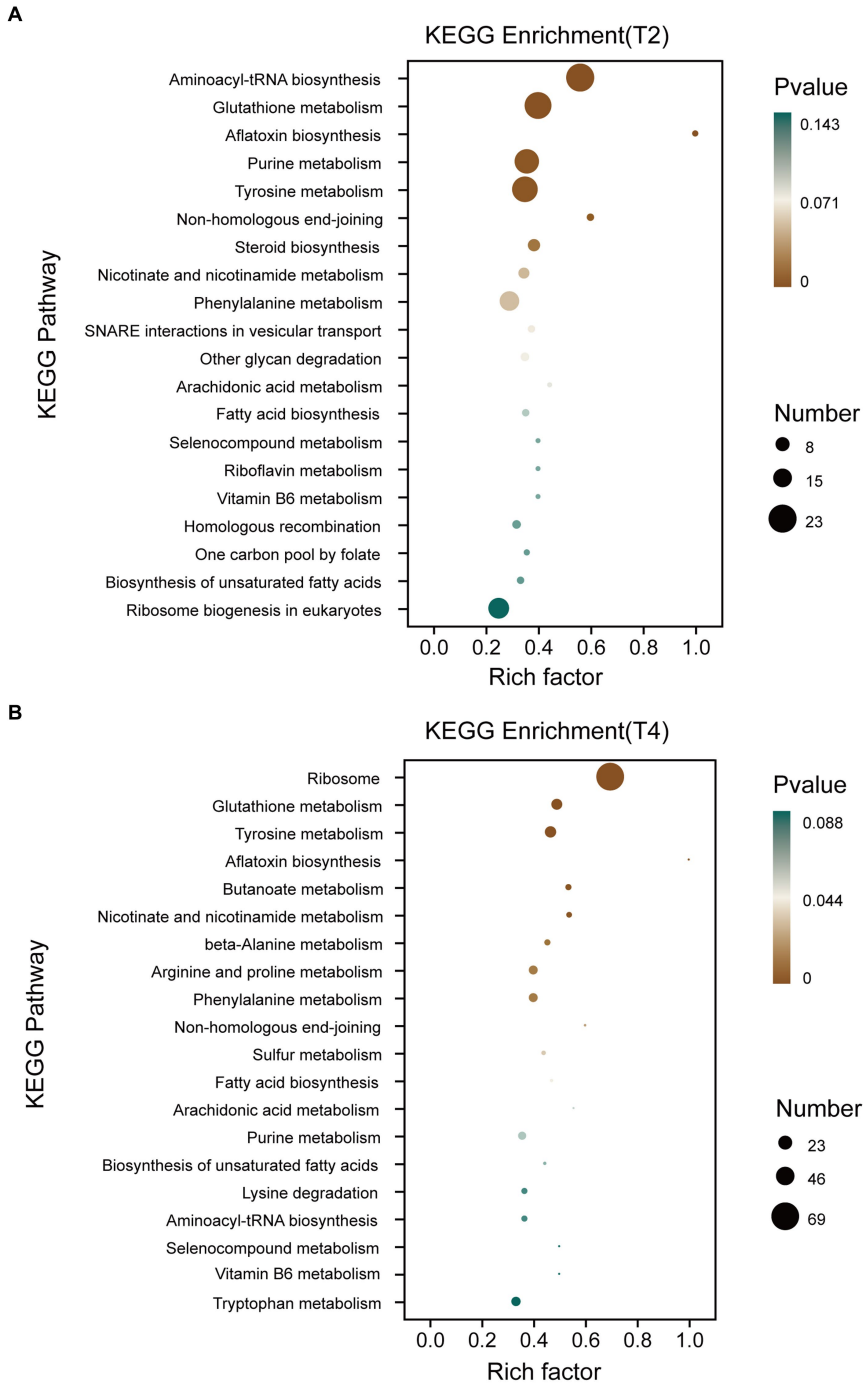
KEGG enrichment classification of differentially expressed genes under different treatments. **(A)** KEGG enrichment classification of differentially expressed genes treated with T2. **(B)** KEGG enrichment classification of differentially expressed genes treated with T4.

The Kyoto Encyclopedia of Genes and Genomes (KEGG) pathway database encompasses molecular interaction networks within each biological pathway, along with the distinctive adaptations of organisms. These resources serve to elucidate the primary biological functions carried out by DEGs. KEGG pathway analysis was conducted on qt40003 after treatment under T2 and T4 conditions (Figure 5). This revealed the enrichment of 699 and 932 DEGs for T2 and T4, respectively, across 20 KEGG pathways. Furthermore, within 12 specific pathways, there were 91 and 173 genes exhibiting significant enrichment. Notably, in T2, these pathways included aminoacyl-tRNA biosynthesis, glutathione metabolism, aflatoxin biosynthesis, purine metabolism, tyrosine metabolism, and non-homologous end joining, while in T4, they included the ribosome, glutathione metabolism, tyrosine metabolism, aflatoxin biosynthesis, butanoate metabolism, nicotinate and nicotinamide metabolism, beta-alanine metabolism, and beta-alanine.

### 3.6 Analysis of genes related to salt stress

**Figure 6:**
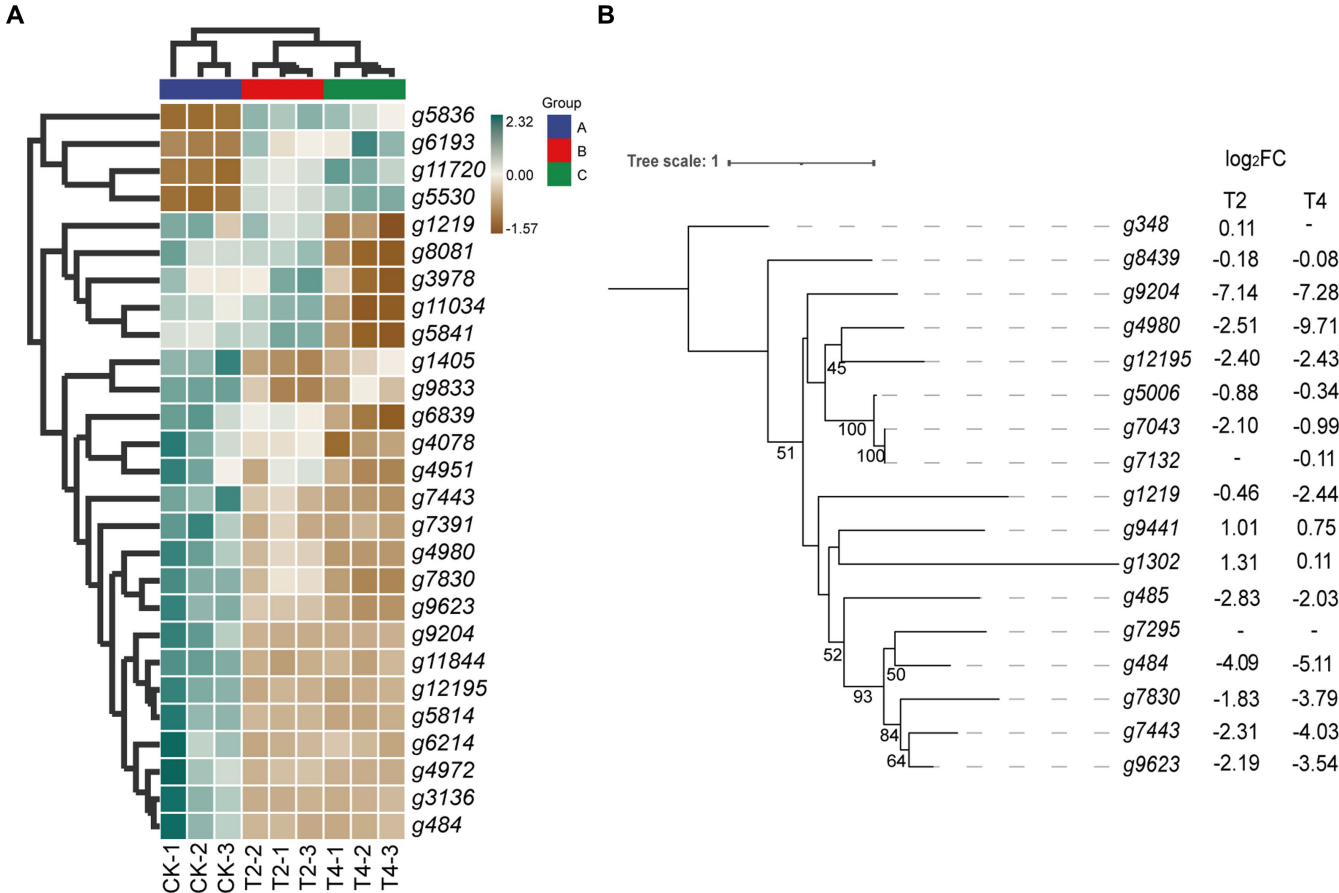
Relationship diagram of differentially expressed genes in the glutathione metabolic pathway. **(A)** Heat map of differential expression genes of glutathione metabolic pathways. **(B)** Phylogenetic tree of glutathione transferase gene family showing its evolution. Bootstrap values (*n* = 1,000).

The KEGG metabolic pathway annotation results under T2 and T4 treatments revealed that the most significantly enriched metabolic pathway was glutathione metabolism. Consequently, we conducted a screening of differentially expressed genes within the glutathione metabolism pathway (Figure 6A). The results showed that four genes, namely *g5836*, *g6193*, *g11720*, and *g5530*, exhibited an upregulation in expression with increasing NaCl concentration. Five genes—*g1219*, *g8081*, *g3978*, *g11034*, and *g5841*—displayed no significant changes in expression levels under T2 conditions, while their expression was significantly downregulated under T4 conditions. In contrast, the expression levels of the remaining 13 genes decreased under both T2 and T4 treatments, with nine of them belonging to the glutathione transferase family (Table 3).

**Table 3:** Functional description of differentially expressed genes of glutathione metabolic pathway.

Gene id	Description
*g5836*	Belongs to the spermidine spermine synthase family
*g6193*	Gamma-glutamyltranspeptidase
*g11720*	Belongs to the Orn Lys Arg decarboxylase class-II family
*g5530*	Ribonucleotide reductase, small chain
*g1219*	Glutathione S-transferase, C-terminal domain
*g8081*	Belongs to the class-I pyridine nucleotide-disulfide oxidoreductase family
*g3978*	Catalyzes the oxidative decarboxylation of 6- phosphogluconate to ribulose 5-phosphate and CO(2)
*g11034*	Gamma-glutamyltranspeptidase
*g5841*	Glutamate-cysteine ligase
*g1405*	Brf1-like TBP-binding domain
*g9833*	Gamma-glutamyltranspeptidase
*g6839*	Glutathione S-transferase, N-terminal domain
*g4078*	Glutathione S-transferase, N-terminal domain
*g4951*	Glutathione S-transferase, N-terminal domain
*g7443*	Belongs to the GST superfamily
*g7391*	Glutathione S-transferase, C-terminal domain
*g4980*	Glutathione S-transferase, C-terminal domain
*g7830*	Belongs to the GST superfamily
*g9623*	Belongs to the GST superfamily
*g9204*	Glutathione S-transferase, C-terminal domain
*g11844*	Belongs to the glutathione peroxidase family
*g12195*	Glutathione S-transferase, C-terminal domain
*g5814*	Aldo/keto reductase family
*g6214*	Glutathione S-transferase, C-terminal domain
*g4972*	Glutathione S-transferase
*g3436*	Saccharopine dehydrogenase NADP binding domain
*g484*	Glutathione S-transferase, C-terminal domain

Thus, the glutathione transferase family constitutes a significant proportion of these differentially expressed genes. Therefore, we focused on screening for differentially expressed genes within the glutathione transferase family. The results show that, apart from the upregulation of *g9441* and *g1302*, the expression levels of all genes under both T2 and T4 conditions were significantly downregulated. Eight genes (*g9204*, *g4980*, *g12195*, *g1219*, *g484*, *g7830*, *g7443*, and *g9623*) exhibited more pronounced downregulation with increasing salt concentration. Subsequently, phylogenetic tree analysis (Figure 6B) revealed significant homology between two of the upregulated genes (*g9441* and *g1302*).

**Figure 7:**
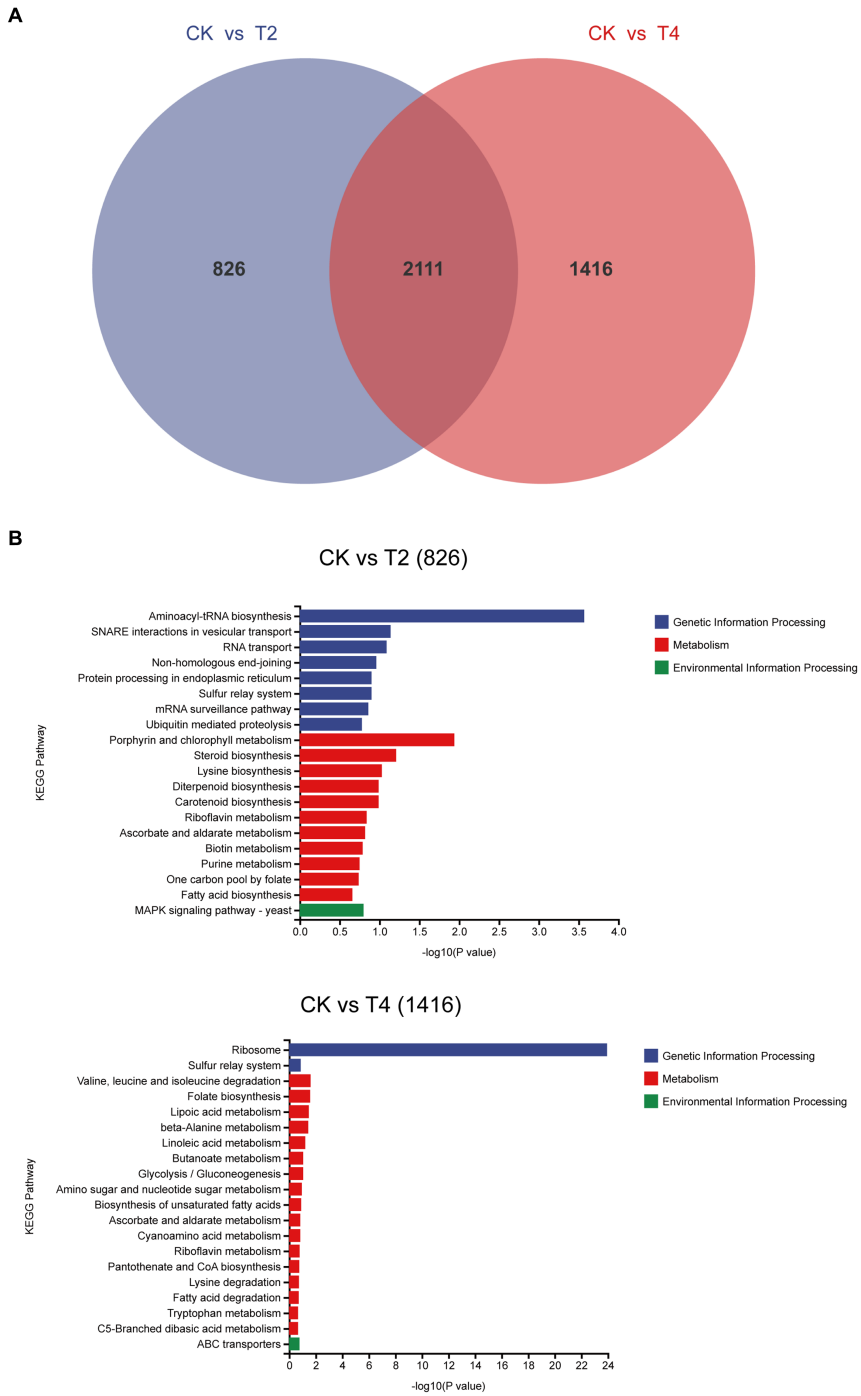
Analysis of non-overlapping differentially expressed genes across distinct treatments. **(A)** Venn diagram of differentially expressed genes under T2 and T4 treatments. **(B)** KEGG metabolic pathway annotation of non-overlapping differentially expressed genes under different treatments.

We identified a shared set of 2,111 differentially expressed genes between the two treatments. Additionally, 826 and 1,416 genes exhibited unique differential expression in T2 and T4, respectively (Figure 7). Subsequently, we conducted KEGG metabolic pathway annotation for these additional genes. The differentially expressed genes in the two treatments were significantly enriched in the porphyrin and chlorophyll metabolic pathway and the valine, leucine, and isoleucine degradation metabolic pathway, respectively. Specifically, five differential genes associated with porphyrin and chlorophyll metabolism in T2 were predominantly upregulated (4/5), with the sole downregulated gene being *g11108* (coproporphyrinogen III oxidase). In contrast, the genes involved in valine, leucine, and isoleucine degradation in T4 were mainly downregulated (10/11), with the only upregulated gene being *g5537* (AMP-binding enzyme) (Table 4).

**Table 4:** Genes involved in the significantly enriched metabolic pathways of non-overlapping genes in T2 and T4 treatments.

	Gene Id	Log_2_FC	Functional description
T2	g11979	1.10	Belongs to the class-I aminoacyl-tRNA synthetase family
	g11108	−1.25	Coproporphyrinogen III oxidase
	g3692	1.07	Belongs to the class-I aminoacyl-tRNA synthetase family
	g5198	1.92	short-chain dehydrogenase
	g1609	1.02	Porphobilinogen deaminase, dipyromethane cofactor binding domain
T4	g811	−1.98	Belongs to the aldehyde dehydrogenase family
	g4650	−2.07	Belongs to the 3-hydroxyisobutyrate dehydrogenase family
	g5537	3.95	AMP-binding enzyme
	g11905	−1.27	Belongs to the enoyl-CoA hydratase isomerase family
	g8039	−1.28	Aldehyde dehydrogenase family
	g3935	−1.12	It is involved in ketone body synthesis and cholesterol synthesis
	g1401	−1.10	Belongs to the class-IV pyridoxal-phosphate-dependent aminotransferase family
	g569	−1.08	Belongs to the class-III pyridoxal-phosphate-dependent aminotransferase family
	g3500	−3.17	Aldehyde dehydrogenase family
	g9014	−1.04	Thiolase, N-terminal domain
	g12065	−1.755	AMP-binding enzyme C-terminal domain

The effect of NaCl on cell growth is mainly caused by oxidative damage to the biofilm and the denaturation of nucleic acids and proteins caused by reactive oxygen species (ROS) free radicals that are generated, resulting in cell death. Therefore, to reduce ROS damage, an antioxidant system consisting of antioxidant enzymes and non-oxidative enzymes is essential (Xiang et al., 2019). We conducted further screening of 13 active oxygen scavenging genes, including peroxidase, glutathione peroxidase, and catalase. Under T4 treatment, we identified six upregulated and seven downregulated ROS scavenging genes, including peroxidase and catalase. Additionally, we found five DEGs related to the cell wall or extracellular structure, all of which were hydrophobic fungal proteins. Of these, *g2257* and *g10606* exhibited a high level of expression (Table 5).

**Table 5:** The predicted genes related to osmotic protection and cell wall of qt40003 under NaCl stress.

	Gene id	T4(log_2_FC)	Function description
Cell wall	*g2257*	4.311234	Fungal hydrophobin
	*g10606*	8.41909	Fungal hydrophobin
	*g12011*	0.858408	Fungal hydrophobin
	*g8412*	−1.38319	Fungal hydrophobin
	*g10244*	0.710579	Fungal hydrophobin
Scavenging	*g1424*	−1.1314	Peroxidase catalase subfamily
	*g11844*	−1.32796	Belongs to the glutathione peroxidase family
	*g8864*	−5.06323	Belongs to the peroxidase familyactivity
	*g10787*	−0.82274	Peroxidase
	*g1127*	−0.31405	Belongs to the peroxidase family
	*g3402*	1.042145	Belongs to the peroxidase family
	*g10014*	0.543035	Belongs to the peroxidase family
	*g5387*	−1.09096	Peroxidase
	*g9374*	0.474377	Peroxidase
Ros	*g594*	1.692462	Catalase
	*g7039*	0.790193	Catalase
	*g4507*	−0.7587	Catalase
	*g12119*	0.591896	Catalase

### 3.7 Validation of the RNA-seq results by qPCR

**Figure 8:**
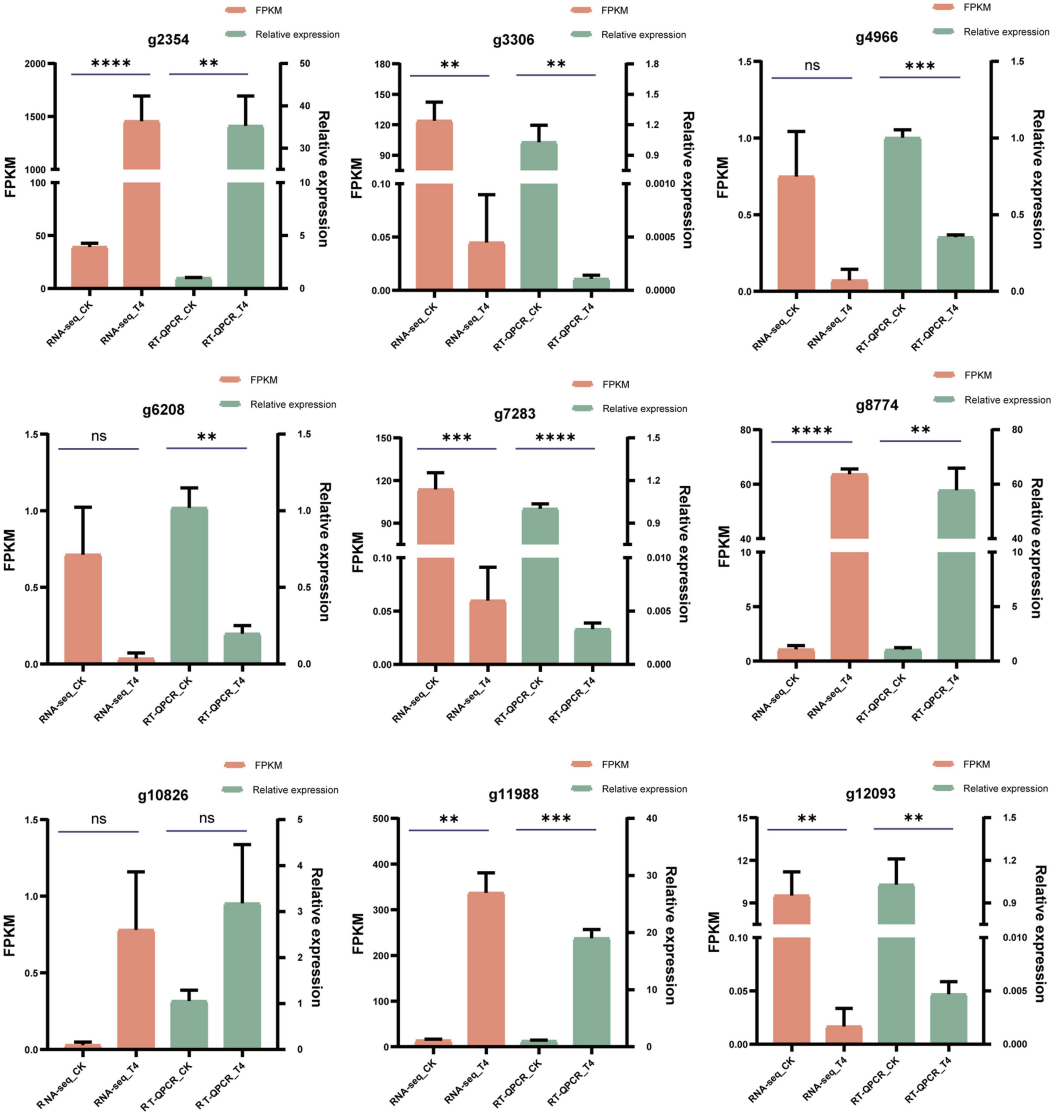
qPCR validation of differentially expressed genes. The reliability of RNA-seq results for 10 differentially expressed genes was substantiated through qPCR validation. FPKM values from the RNA-seq analysis were presented in the orange column, while qPCR measurements were displayed in the green column. The data were expressed as the mean ± standard error (*n* = 3). In the context of an unpaired *t*-test, “*” signified a significance level of *p* < 0.05, “**” indicated *p* < 0.01, “***” denoted *p* < 0.001, “****” represented an exceptionally profound significance, and “ns” indicated a lack of statistical significance.

To validate the accuracy of the RNA-seq findings, nine genes showing substantial expression changes under T4 conditions were selected from the transcriptomic data, including four upregulated and five downregulated genes. These expression changes were then verified by quantitative PCR (qPCR). Using custom-designed primers (Table 6), qPCR was used for the precise quantification of the expression changes across these nine genes in the CK and T4 groups. This showed a slight variance between the qPCR and RNA-seq results for the differentially expressed genes (Figure 8), but the overall trends were in agreement, confirming the reliability of the RNA-seq results.

**Table 6:** Primer sequences used for qPCR analysis.

Gene	Forward (5′-3′)	Reverse (5′-3′)
*g2354*	CGAGGCAGTAGGCTTCCATTTTC	CGAGGCAGTAGGCTTCCATTTTC
*g7283*	GCCAAGACTCAACATCCATACG	GCCTTCACCTTCAACGACCAC
*g3306*	TCCTATTTCGTATTCTTCCACCCG	GGTAATGCCTGATCCCTTTTG
*g10826*	TTTCCCATTGAGAAGGTCCAGG	TTCGCTACCGCTAAGACGCC
*g6208*	TCGCCTCCCTCTTCTCCAAC	GAAAGTGTCCACAAGCCCCTC
*g4966*	GCCACATCTCAGAATACAACAACG	GACCGCAGAAATCAGCCAAG
*g12093*	CTATGGCGTCTTCGGTCTCAC	GGATCTTTGGAAACTGCCCTCTT
*g11988*	TCGGCAACGGCACATCAGCAG	GCTTCGCTCAACAAAGGCATAC
*g8774*	CTGTCGCTTCTTGTCCTCCG	TGGTGAGCATCTGGCTGTAGTC
*Tubulin*	GCTACCTGACCTGCTGCTCT	TCGCCGACACGCTTGAACAG

## 4 Discussion

Adverse conditions can cause varying degrees of damage to microorganisms. Currently, research on microbial environmental stress primarily focuses on heavy metals, PAHs (polycyclic aromatic hydrocarbons), organic acids, and other factors (Xiang et al., 2019). *TH* is one of the potent biocontrol fungi today, and studying its salt tolerance is particularly necessary. However, the response mechanisms of fungi to salt stress are highly complex.

Research on *Trichoderma* spp. has accelerated considerably with the availability of fully sequenced genomes. Plant biotechnology will also benefit from *Trichoderma* genome mining (Mukherjee et al., 2013). Rosolen et al. used genome-wide phylogenetic analysis to elucidate the relationships between newly sequenced species and other *Trichoderma* species (Mukherjee et al., 2013). Wang et al. conducted a genomic analysis of *TH*, revealing the biosynthetic diversity of its specialized secondary metabolites (Al-Salihi and Alberti, 2023). Fanelli et al. presented the genome of the *TH* strain ITEM908, a biocontrol agent. Through genomic analysis of ITEM908, they identified the occurrence and correlation of genes associated with biocontrol and slope (Fanelli et al., 2018). Vieira et al. were the first to establish a method for *TH* genome editing. The mutants generated, produced by the overexpression of Cas9 and nutritional markers, serve as tools for functional analysis of biocontrol genes, selection of biocontrol strains, and the generation of novel strains for biotechnological applications (Vieira et al., 2021). Here, for the first time, we have assembled the complete genome sequence (44.47 Mb) of *TH* qt40003, providing a foundational resource for future in-depth exploration of this biocontrol fungus. This comprehensive genomic resource opens new avenues for understanding the biology, evolutionary dynamics, and functional genomics of *TH*. The elucidation of the entire genome provides valuable insights into the genetic basis of its biocontrol capabilities and offers opportunities for the development of innovative strategies for sustainable agriculture, disease management, and biotechnological applications. The availability of this high-quality genome sets the stage for further investigations into the molecular mechanisms underlying the biocontrol activities of *TH* and provides a solid foundation for harnessing its potential in diverse fields.

Transcriptome sequencing based on cDNA using second-generation high-throughput sequencing technology has higher detection throughput and a broader detection range than gene chip technology (Liu et al., 2015). In recent years, this technology has been widely applied for research on fungal functional gene discovery and metabolic regulation. To ensure sufficient fungal biomass in this study, we used second-generation high-throughput sequencing technology to conduct RNA-seq on the qt40003 strain under NaCl stress conditions at 0, 2, and 4% NaCl concentrations, with three biological replicates for each condition. It is of note that previous studies have shown significant differences in the salt tolerance response of microorganisms to low and high salt levels (Grada and Weinbrecht, 2013). Therefore, we screened for differentially expressed genes under two treatment concentrations, and obtained a total of 2,937 and 3,527 genes for 2 and 4% NaCl, respectively. T4 conditions resulted in 590 more differentially expressed genes than T2 conditions. Furthermore, we observed that the fold change in the expression of many genes also varied with the increase in NaCl concentration. Therefore, the selection of an appropriate NaCl concentration is crucial for studying the salt tolerance mechanisms of *TH*. The functional enrichment analysis of these differentially expressed genes revealed associations with cellular processes, cellular components, metabolic processes, and other functions. This result is consistent with the experimental research results of Wang et al. on fungal stress (Wang, 2017). In the GO enrichment analysis, differentially expressed genes are categorized into biological processes, molecular functions, and cellular components. Under T2 and T4 treatments, significant enrichments in biological processes are observed in “tRNA aminoacylation for protein translation” and “cytosolic ribosome.” Molecular functions show prominent enrichments in “aminoacyl-tRNA ligase activity” and “cytoplasmic translation,” while cellular components exhibit significant enrichments in “intrinsic component of external side of plasma membrane” and “structural constituent of ribosome.” In the KEGG pathway analysis, differentially expressed genes are mainly classified into two major categories: “Genetic Information Processing” and “Metabolism.” Under T2 and T4 treatments, genes related to Genetic Information Processing significantly enrich in “Aminoacyl-tRNA biosynthesis” and “Ribosome,” while those associated with Metabolism predominantly enrich in “Glutathione metabolism.” Under the two treatment conditions, differentially expressed genes exhibit distinct patterns in GO and KEGG enrichment analyses. This suggests that the fungus may adopt different biological responses and metabolic pathway regulation strategies to adapt to changes in salt concentration under different salt levels. However, in the KEGG metabolic pathways, both treatments significantly enriched in the glutathione metabolism pathway. Therefore, we focused on analyzing differentially expressed genes related to glutathione metabolism.

Glutathione S-transferase (GST) enzymes are present in many evolved organisms and are essential for the defense against reactive oxygen species (Muslu et al., 2023). GSTs play a role in the development of defenses against biotic and abiotic challenges, especially in defending plants from various stresses such as drought, salinity, and heavy metal exposure, and have diverse functions, including the production of oxylipins as precursors to jasmonic acid. GSTs help eliminate cytotoxic or genotoxic components in cells that can damage DNA, RNA, or proteins (Noctor et al., 2002). In our KEGG pathway enrichment analysis, we screened for differentially expressed genes in the glutathione metabolism pathway and found that the expression of GST decreased with increasing NaCl concentration, consistent with results reported by Muslu et al. (2023) and others. The inhibition rate of qt40003 mycelium growth at 2% NaCl concentrations was only 11.75%, while the inhibition rate at 4% NaCl reached 60.38%, indicating that the salt tolerance of qt40003 was significantly reduced at this concentration, which we speculated might be related to the downregulation of GST. Unlike our study, Xiang et al., in their investigation of salt stress in *TH*, revealed through enrichment analysis of KEGG metabolic pathways that, under 0.6 mol/L salt stress, the most significantly downregulated pathway in *TH* is the biosynthesis of amino acids (Xiang et al., 2019). They used a concentration of 0.6 mol/L in their study, while in our research, the concentration was 4%. This difference in concentration may lead to notable variations in the observed biological responses between the two studies. In our study, we observed that, at a concentration of 4% NaCl, downregulated genes in the *TH* transcriptome were primarily enriched in the glutathione metabolism pathway, indicating a cellular preference for addressing oxidative stress and detoxification reactions. In contrast, at 0.6 mol/L concentration in Xiang et al.’s study, the differentially expressed genes, particularly downregulated ones, were predominantly enriched in amino acid metabolism pathways. This observation suggests a potential emphasis on regulating amino acid metabolism to maintain fundamental biological functions under higher salt concentrations. Glutathione metabolism is primarily involved in cellular detoxification and antioxidant responses, while amino acid metabolism plays a crucial role in various biological processes. Amino acids serve as the building blocks for protein synthesis, provide energy through metabolic pathways, supply carbon sources, and contribute significantly to immune responses (Ling et al., 2023). Therefore, under 0.6 mol/L salt stress, *TH* may prioritize the regulation of amino acid metabolism to sustain basic biological functions. On the other hand, at 4% concentration, there appears to be a heightened emphasis on defense against oxidative stress, as evidenced by the significant enrichment in glutathione metabolism. This difference likely reflects the varying responses and physiological adaptations of *TH* to stress under different salt concentrations.

High NaCl concentrations cause osmotic stress on cells, and can also lead to the accumulation of excessive reactive oxygen species in cells, triggering the free radical chain reaction of membrane lipid peroxidation, producing excessive harmful free radicals and aggravating the toxicity on cells. In this study, 13 genes related to active oxygen clearance were detected under 4% NaCl stress, including peroxidase, glutathione peroxidase, and catalase, and 10 of these genes were upregulated, indicating that the qt40003 antioxidant oxidase system can actively respond to oxidative damage caused by salt stress.

Fungal hydrophobic proteins are low-molecular-weight amphoteric proteins secreted by higher filamentous fungi with special physical and chemical properties, which play an important role in the growth and development of fungi and environmental communication (Wösten and Wessels, 1997). Hydrophobic proteins typically play a role in stabilizing membrane structures, and their high expression may contribute to maintaining the integrity of cellular membranes, thereby slowing down or alleviating the damage caused by salt stress to the cell membrane. Hydrophobic proteins may be involved in counteracting oxidative stress induced by salt stress. Under adverse conditions, organisms may produce an excess of reactive oxygen species, and the high expression of hydrophobic proteins may aid in clearing these harmful substances, protecting cells from oxidative damage. *Trichoderma* species contain a high diversity of hydrophobic proteins (Kubicek et al., 2008). Huang et al. found that expression of the *T. asperellum* ACCC30526 class II hydrophobic protein gene *HFB2-6* was induced under carbon and nitrogen stress, and may be involved in plant rhizosphere colonization (Huang et al., 2015). We screened five fungal hydrophobic proteins under 4% NaCl stress, and two hydrophobic proteins were highly induced.

The current research provides valuable insights into the physiological and molecular response mechanisms of *TH* under salt-stress conditions. However, there are still many future research directions that will further expand our understanding of this field. Deeper research is needed into the differentially expressed genes identified herein to determine their exact functions in the salt-stress response. This may involve gene knockout or overexpression experiments to validate their roles in salt tolerance. In addition to gene-level research, future studies should focus on understanding the changes to proteins and metabolites under salt-stress conditions. This will help to reveal a more comprehensive response mechanism.

In summary, research on the salt-stress response of *TH* provides profound insights and has the potential to positively impact fields such as agriculture, ecology, and biotechnology. Future research will contribute to unlocking more potential applications and ecosystem effects, better addressing the challenges posed by salt stress.

## 5 Conclusion

The complete genome sequence of *TH* strain qt40003, which is 44.47 Mb in length, is reported here for the first time. Under NaCl stress, a concentration of 10% was the lethal threshold for qt40003. On transcriptomic analysis, 2,937 and 3,527 differentially expressed genes were obtained in control vs. 2% NaCl and control vs. 4% NaCl, respectively. The expression of GST was significantly inhibited on 4% NaCl treatment, and we speculate that the expression of GST is regulated by the salt stress response pathway. The decline in GST expression may be an attempt to allocate resources to other, more urgent survival strategies while responding to salt stress. In this case, the decline in GST may be the result of a cellular stress response that could make organisms more vulnerable to damage in high-salt environments, and so may be related to the decreased tolerance of this strain in salt-stress conditions.

## Data availability statement

The datasets presented in this study can be found in online repositories. The names of the repository/repositories and accession number(s) can be found in the article/Supplementary material.

## Author contributions

QY: Data curation, Investigation, Methodology, Software, Validation, Visualization, Writing – original draft, Writing – review & editing. ZM: Project administration, Resources, Supervision, Writing – review & editing. YH: Resources, Writing – review & editing. SZ: Investigation, Software, Writing – review & editing. JZ: Supervision, Writing – review & editing. YanL: Supervision, Writing – review & editing. YY: Supervision, Writing – review & editing. BX: Project administration, Supervision, Writing – review & editing. JL: Funding acquisition, Methodology, Project administration, Resources, Supervision, Writing – review & editing. YanlL: Resources, Supervision, Validation, Writing – review & editing.
