# Supplementary material for: Genome-wide transcriptome profiling reveals molecular response pathways of Trichoderma harzianum in response to salt stress
Source: Front Microbiol. 2024 Feb 1;15:1342584. doi: 10.3389/fmicb.2024.1342584 (PMC10867199; doi:10.3389/fmicb.2024.1342584)
Supplement: Supplementary file 1 [file Data_Sheet_1.PDF]

Supplementary Material.

1 Supplementary Figures and Tables

1.1 Supplementary Figures

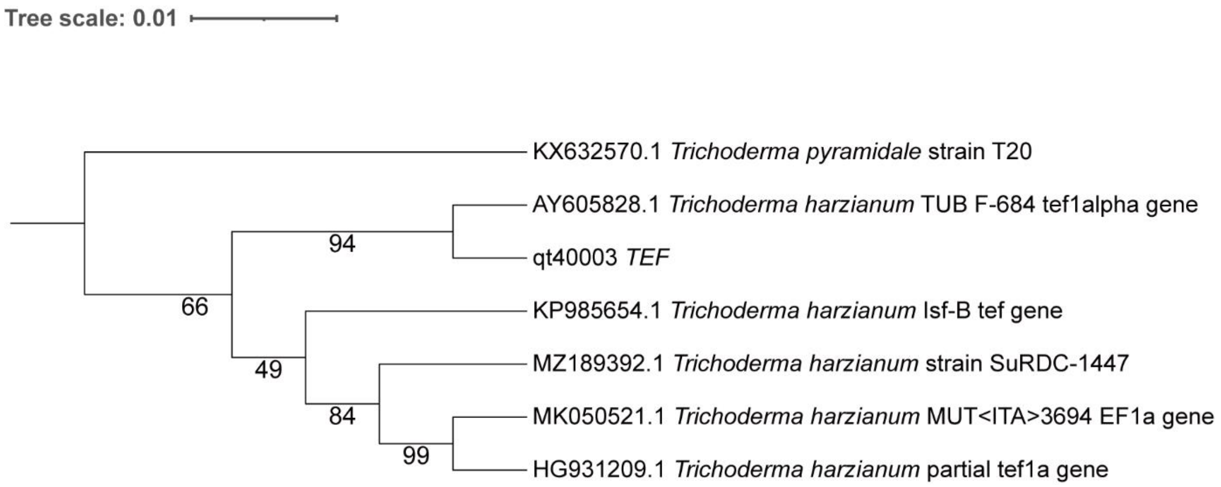

Figure.S1 Phylogenetic tree based on *TEF* sequences of qt40003 and six other different *Trichoderma* species.

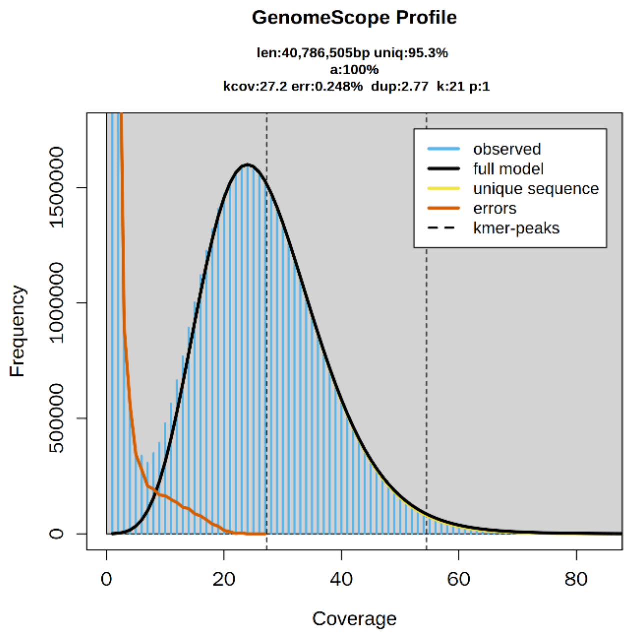

Figure.S2 qt40003 K-mer depth distribution

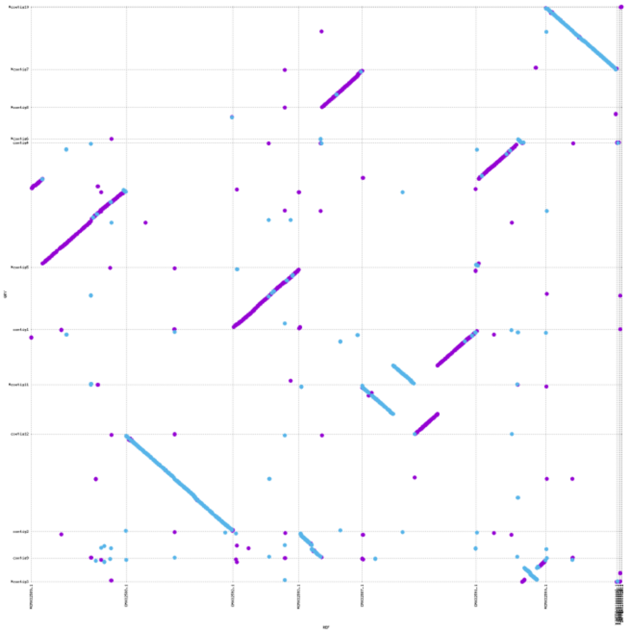

Figure.S3 The syntenic relationships between qt40003 and CDMCC_20739. Y-axis represents qt40003 contigs, and x-axis represents CDMCC_20739 genome. The purple dot or line represents forward matches, and the blue dot or line represents reverse matches between two genomes.

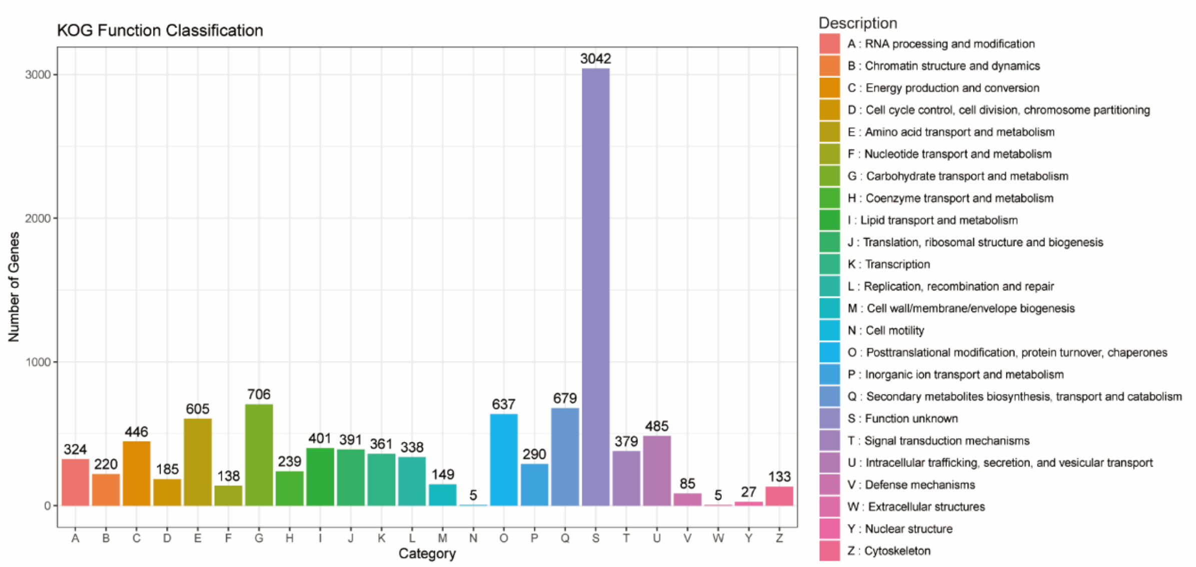

Figure.S4 qt40003KOG functional classification diagram

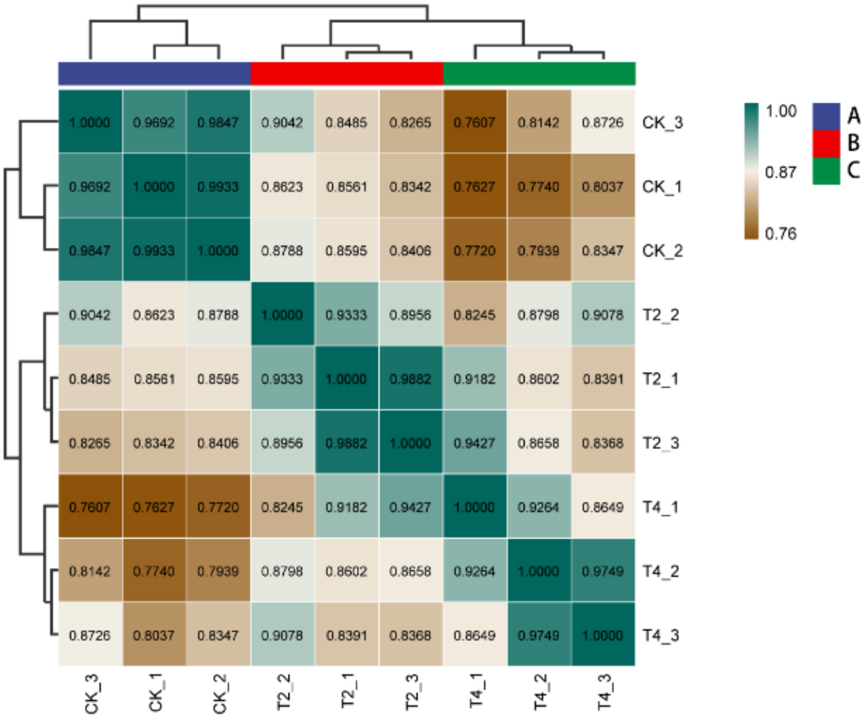

Figure.S5 Heat map of correlation between samples

1.2 Supplementary Tables

Table S1 Classification of Repetitive Sequences qt40003

Class	Count	bpMasked	%masked
DNA	--	--	--
Academ-2	1	207	0.00%
CMC-EnSpm	4	654	0.00%
Crypton-S	2	825	0.00%
Dada	1	444	0.00%
IS3EU	2	1170	0.00%

Kolobok-H	14	8757	0.02%
MITE	459	86367	0.19%
MULE-MuDR	36	15717	0.04%
P	8	1524	0.00%
PIF-Harbing	3	468	0.00%
PIF-ISL2EU	2	387	0.00%
PiggyBac	1	42	0.00%
PiggyBac-X	1	129	0.00%
TcMar-Ant1	49	16282	0.04%
TcMar-Fot1	10	5289	0.01%
TcMar-Marin	2	387	0.00%
TcMar-Pogo	2	738	0.00%
TcMar-Tc1	2	1203	0.00%
Zisupton	12	2982	0.01%
hAT-Ac	11	2531	0.01%
hAT-Charlie	1	189	0.00%
hAT-Restles	119	78497	0.18%
hAT-Restless	7	1577	0.00%
hAT-Tip100	2	273	0.00%
hAT-hobo	2	456	0.00%

LINE	--	--	--
CR1	5	1122	0.00%
CRE	1	261	0.00%
CRE-Ambal	1	129	0.00%
I	2	237	0.00%
I-Jockey	1	252	0.00%
L1	8	1791	0.00%
L1-Tx1	3	903	0.00%
L2	3	753	0.00%
Penelope	2	270	0.00%
R1	1	351	0.00%
R2	1	114	0.00%
R2-NeSL	1	534	0.00%
RTE-BovB	2	480	0.00%
Rex-Babar	1	354	0.00%
Tad1	387	545399	1.23%
LTR	--	--	--
Copia	40	6669	0.01%
ERV-Foamy	1	132	0.00%
ERV1	4	1068	0.00%
ERVK	2	447	0.00%

Gypsy	1473	2164981	4.87%
Pao	22	4749	0.01%
RC	--	--	--
Helitron	39	13722	0.03%
Unknown	1471	942616	2.12%

Total interspersed	4224	3914429	8.80%
TandemRepeat	7293	357908	0.80%
Total	11517	4272337	9.61%

Table S2 BUSCO evaluation statistics

Category	Fungi_odb10
Complete BUSCOs (C)	739(97.5%)
Complete and single-copy BUSCOs (S)	736(97.1%)
Complete and duplicated BUSCOs (D)	3(0.4%)
Fragmented BUSCOs (F)	4(0.5%)
Missing BUSCOs (M)	15(2.0%)
Total BUSCO groups searched	758

Table S3 Effects of different NaCl stress on physiological indexes of 40003 mycelia

Sample	Clean reads	Total mapped reads	Mapped ratio%
CK	55,358,744	54,984,347	99.32%
	54,638,214	54,263,850	99.31%
	45,090,030	44,758,330	99.26%
T2	35,403,582	35,167,834	99.33%
	40,382,818	40,099,251	99.30%
	42,931,066	42,660,409	99.37%
T4	45,502,518	45,183,029	99.30%
	48,569,900	48,281,691	99.41%
	49,077,086	48,755,660	99.35%
